# Interaction of a dengue virus NS1‐derived peptide with the inhibitory receptor KIR3DL1 on natural killer cells

**DOI:** 10.1111/cei.12722

**Published:** 2015-11-24

**Authors:** E. Townsley, G. O'Connor, C. Cosgrove, M. Woda, M. Co, S. J. Thomas, S. Kalayanarooj, I.‐K. Yoon, A. Nisalak, A. Srikiatkhachorn, S. Green, H. A. F. Stephens, E. Gostick, D. A. Price, M. Carrington, G. Alter, D. W. McVicar, A. L. Rothman, A. Mathew

**Affiliations:** ^1^ Division of Infectious Diseases and ImmunologyUniversity of Massachusetts Medical SchoolWorcesterMAUSA; ^2^ Cancer and Inflammation Program, Laboratory of Experimental ImmunologyLeidos Biomedical Research Inc., Frederick National Laboratory for Cancer ResearchFrederickMDUSA; ^3^ Ragon Institute at MGH, MIT And HarvardMassachusetts General Hospital, Harvard Medical SchoolBostonMAUSA; ^4^ Walter Reed Army Institute of ResearchSilver SpringMDUSA; ^5^ Queen Sirikit National Institute for Child HealthBangkokThailand; ^6^ Department of VirologyArmed Forces Research Institute of Medical SciencesBangkokThailand; ^7^ Centre for Nephrology and the Anthony Nolan TrustRoyal Free Campus, University CollegeLondonUK; ^8^ Cardiff University School of MedicineInstitute of Infection and ImmunityCardiffUK; ^9^ Human Immunology Section, Vaccine Research Center, National Institute of Allergy and Infectious Diseases, National Institutes of HealthBethesdaMDUSA; ^10^ Institute for Immunology and Informatics, University of Rhode IslandProvidenceRIUSA

**Keywords:** dengue, HLA, KIR, NK, pathogenesis

## Abstract

Killer immunoglobulin‐like receptors (KIRs) interact with human leucocyte antigen (HLA) class I ligands and play a key role in the regulation and activation of NK cells. The functional importance of KIR–HLA interactions has been demonstrated for a number of chronic viral infections, but to date only a few studies have been performed in the context of acute self‐limited viral infections. During our investigation of CD8^+^ T cell responses to a conserved HLA‐B57‐restricted epitope derived from dengue virus (DENV) non‐structural protein‐1 (NS1), we observed substantial binding of the tetrameric complex to non‐T/non‐B lymphocytes in peripheral blood mononuclear cells (PBMC) from a long‐standing clinical cohort in Thailand. We confirmed binding of the NS1 tetramer to CD56^dim^ NK cells, which are known to express KIRs. Using depletion studies and KIR‐transfected cell lines, we demonstrated further that the NS1 tetramer bound the inhibitory receptor KIR3DL1. Phenotypical analysis of PBMC from HLA‐B57^+^ subjects with acute DENV infection revealed marked activation of NS1 tetramer‐binding natural killer (NK) cells around the time of defervescence in subjects with severe dengue disease. Collectively, our findings indicate that subsets of NK cells are activated relatively late in the course of acute DENV illness and reveal a possible role for specific KIR–HLA interactions in the modulation of disease outcomes.

## Introduction

Killer immunoglobulin‐like receptors (KIRs) are expressed predominantly on natural killer (NK) cells and interact with specific human leucocyte antigen (HLA) class I ligands to transduce inhibitory or activating signals 1. One of the best‐characterized and highly polymorphic members of the KIR family is the inhibitory receptor KIR3DL1, which is present in >90% of the human population and has at least 62 allotypes 2. Interactions between KIR3DL1 and the HLA‐Bw4 motif act to maintain natural killer (NK) cell inhibition. However, the down‐regulation of major histocompatibility complex (MHC) class I molecules that often follows viral infection or cellular transformation alleviates NK cell inhibition via KIR3DL1, leading to proinflammatory cytokine release and cytolytic activity. A role for KIR3DL1 in the control of chronic viral infections has been proposed on the basis of associations with disease outcome in HIV‐infected individuals 3, 4, 5, 6, 7, 8. These studies suggest that both MHC class I and KIR genotypes may contribute to protection in the context of HLA‐B57. Moreover, KIRs that interact with HLA‐C have been linked epidemiologically to the development of liver disease in hepatitis C virus (HCV)‐infected patients and protection from HCV infection in a cohort of intravenous drug users 9. In contrast, the role of KIR‐HLA interactions in acute self‐limited viral infections remains largely unexplored.

Dengue virus (DENV) is a member of the flavivirus family comprising at least four distinct serotypes. Transmitted by the mosquito *Aedes aegypti*, DENV is endemic in the tropics/subtropics and causes an acute febrile illness known as dengue fever (DF). However, a small percentage of individuals experience a more severe syndrome known as dengue haemorrhagic fever (DHF). The key features of DHF are plasma leakage and a bleeding tendency, which develop as the fever subsides with clearance of viraemia 10, 11. Although both viral and host‐specific factors probably influence clinical outcome, prospective cohort studies have identified secondary infection with a heterologous DENV serotype as a major risk factor for DHF 12. At the mechanistic level, pre‐existing antibodies 13, memory T cell responses 12, 14 and certain HLA genotypes 15, 16, 17, 18 have all been linked with more severe dengue illness.

A number of reports describe associations between HLA class I genotypes and dengue disease severity 15, 16, 17, 18. In one earlier study, extended HLA region haplotypes including tumour necrosis factor (TNF), lymphotoxin alpha (LTA) and lymphotoxin beta (LTB), together with specific combinations of class I and class II alleles, were associated strongly with DHF during secondary DENV infection. Various aspects of disease outcome after DENV exposure have also been linked to functionally defined HLA class I supertypes 19, as well as the MHC class I‐related chains A/B (MICA/B) 20, 21, 22. These latter proteins are up‐regulated in stressed cells and interact with NKG2D, an activating receptor on NK cells. More recently, two small genetic studies evaluated associations between KIR–ligand pairs and susceptibility to dengue in Gabon and Southern Brazil 23, 24. Petitdemange *et al*. found no evidence of a role for KIR genotypes in patients infected with DENV‐2. In contrast, Beltrame *et al*. detected an association between certain KIR genes and their cognate HLA ligands in the context of infection with DENV‐3. Differences in population origin and the infecting DENV serotype may explain these disparate results. Other studies have noted NK cell activation during acute DENV infection. In particular, Azeredo *et al*. linked early activation of NK cells with mild DENV disease 25, whereas Green *et al*. found increased frequencies of NK cells expressing CD69 in children who developed DHF compared to those with attenuated disease 26. The mechanisms by which NK cells contribute to immune protection and immunopathogenesis in DENV infection therefore require further elucidation 27, 28.

We recently characterized antigen‐specific CD8^+^ T cells directed against a highly conserved HLA‐B57‐restricted epitope derived from DENV non‐structural protein‐1 (NS1) 29. In the present study, we examined binding of the corresponding B57‐NS1_26–34_ tetramer (NS1 TET) to enriched NK cell populations from samples obtained prior to, during and up to 1 year after the critical phase of illness (around the time of defervescence) in HLA‐B57^+^ subjects from a clinical cohort in Thailand. Using KIR3DL1^+^ healthy donor peripheral blood mononuclear cells (PBMC), we confirmed that the NS1 TET bound mainly to CD56^dim^ NK cells, which are known to express KIRs 30. We then demonstrated that the NS1 TET bound KIR3DL1. To determine whether there was an association between NK cell activation and dengue disease severity, we analysed PBMC from our HLA‐B57^+^ cohort and found marked activation of NS1 TET^+^ NK‐enriched cells at the critical phase of illness in patients who developed DHF. Our results define a specific interaction between the inhibitory receptor KIR3DL1 and a DENV‐derived CD8^+^ T cell epitope with potential relevance to the immunopathogenesis of dengue disease.

## Materials and methods

### Study subjects and blood samples

The study design for patient recruitment and collection of blood samples has been reported in detail elsewhere 11, 43, 44, 45. Briefly, the enrolled subjects were Thai children aged 6 months to 15 years with acute febrile illnesses (<72 h) diagnosed as DF or DHF according to World Health Organization (WHO) guidelines 46. Serology and virus isolation were used to confirm acute DENV infection, and primary and secondary infections were distinguished on the basis of serological responses 11. For donors undergoing a secondary infection, it was not possible to determine the previous infecting serotype(s). Blood samples were obtained daily during acute illness, once during early convalescence and at various intervals during late convalescence. PBMC were isolated by density gradient centrifugation, cryopreserved and stored at −70°C. Samples were numbered relative to the day of defervescence (designated fever day 0). Serological HLA class I typing was performed as described previously using peripheral blood from immune Thai donors at the Department of Transfusion Medicine, Siriraj Hospital 15, 44. Written informed consent was obtained from each subject and/or his/her parent/guardian prior to study participation. The study was approved by the Institutional Review Boards of the Thai Ministry of Public Health, the Office of the US Army Surgeon General and the University of Massachusetts Medical School (UMMS). For control purposes, PBMC were obtained with informed consent from healthy HLA‐B57^+^ dengue‐naïve volunteers aged > 18 years under approval granted by the UMMS Institutional Review Board.

### Peptide‐MHC tetramers

Peptide‐MHC tetramers (pMHC TETs) were either obtained from the NIAID Tetramer Core Facility or generated in‐house as described previously 47. The following conjugates were used in this study: A2‐E_213–221_ TET‐allophycocyanin (APC), B57‐LF9 TET‐phycoerythrin (PE), B57‐NS1_26–34_ TET‐PE, B57‐NS1_26–34_ TET‐APC, B57‐TW10n TET‐PE and B57‐TW10n TET‐APC.

### Flow cytometry

As described previously 29, cryopreserved PBMC from Thai subjects were thawed and washed in RPMI before resting in RPMI/10% fetal bovine serum (FBS) for 2 h at 37°C. Cells were then washed in phosphate‐buffered saline (PBS) and stained with 1 µl of prediluted (1 : 80) LIVE/DEAD^®^ Green (Molecular Probes, Invitrogen, Waltham, MA, USA). After washing in fluorescence activated cell sorter (FACS) buffer (PBS/2% FBS/0·1% sodium azide), cells were incubated with 0·5–2 µl pMHC TET (1 µg/µl with respect to the monomeric component) for 20 min at 4°C. Pretitrated monoclonal antibodies specific for CD3, CD8, CD14, CD19, CD28 or CD56, CD38, CD45RA, CD57, CD69, CD71 and CCR7 were then added for a further 30 min at 4°C. Monoclonal antibodies specific for CD3, CD14, CD16, CD19, CD56, CD69 and KIR3DL1 were used in a separate panel to identify NK cells. For NS1 TET staining of PBMC from healthy individuals, 1 × 10^7^ cells from KIR3DL1^+^ subjects were washed in PBS and stained with LIVE/DEAD^®^ Green. After washing in FACS buffer, cells were incubated with 2 µl pMHC TET or a KIR3DL1‐specific monoclonal antibody for 20 min at 4°C. Pretitrated monoclonal antibodies specific for CD3, CD14, CD16, CD19, CD56, CD161, NKp30, NKp46 and NKG2D were then added for a further 30 min at 4°C. In all experiments, cells were washed and fixed with BD Stabilizing Fixative™ (BD Biosciences, San Jose, CA, USA). Data were collected using a FACSAria™ flow cytometer (BD Biosciences) and analysed with FlowJo version 10 (TreeStar Inc., Ashland, OR, USA). Details of all monoclonal antibodies used in this study are presented in Supporting information, Table S1.

### KIR3DL1^+^ NK cell depletion and NS1 tetramer staining

PBMC were isolated from KIR3DL1^+^ healthy subjects using standard density gradient centrifugation and depleted of KIR3DL1^+^ cells via magnetic bead separation (Miltenyi Biotec, San Diego, CA, USA). KIR3DL1‐depleted PBMC were washed in FACS buffer and incubated with NS1 TET for 50 min at 4°C. After a further wash in FACS buffer, cells were fixed with 100 µl of prediluted (1 : 4) BD Cytofix (BD Biosciences) and kept at 4°C until acquisition. Flow cytometric data were collected and analysed as described above.

### Binding of pMHC tetramers to KIR3DL1‐transfected cell lines

Detailed analyses of KIR3DL1‐transfected lines were performed as reported elsewhere 33. Briefly, human embryonic kidney (HEK) 293 cells were transfected with FLAG‐tagged constructs of KIR3DL1*001, *005 or *015. An anti‐FLAG monoclonal antibody was used to verify KIR3DL1 expression. Transfected cells were preincubated with 10 µg/µl of the blocking monoclonal antibody DX9 or control immunoglobulin (Ig)G, then stained with 0·25 µl of the NS1 TET or the well‐described LF9 TET, representing a self‐derived peptide complexed with HLA‐B57 that binds KIR3DL1 48.

## Statistical analysis

Comparisons between groups were conducted using the Mann–Whitney rank sum test for non‐normally distributed variables. All statistical analyses were performed using GraphPad Prism (GraphPad Software, San Diego, CA, USA).

## Results

### Binding of the NS1 TET to CD8^–^ cells in PBMC from dengue patients

**Figure 1:**
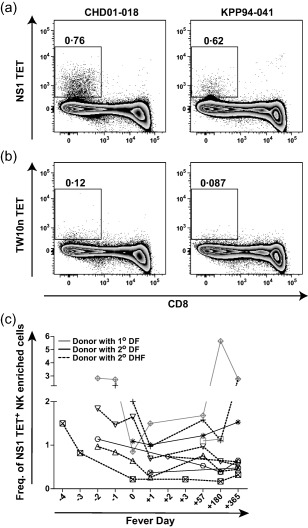
Binding of the NS1 tetramer (TET) to non‐CD8 cells in peripheral blood mononuclear cells (PBMC) from Thai children with dengue. (a,b) Using flow cytometry, frequencies of NS1 TET^+^ (a) and TW10n TET^+^ (b) CD3^–^CD8^–^CD14^–^CD19^–^ [natural killer (NK)‐enriched] cells in PBMC from donors CHD01‐018 and KPP94‐041 at the 1‐year time‐point. (c) Kinetics of NS1 TET^+^ frequencies among NK‐enriched cells during acute dengue illness and convalescence. Fever day 0 indicates the day of defervescence. Symbols distinguish subjects with primary (*n* = 2, grey symbols) *versus* secondary (*n* = 8, black symbols) dengue virus (DENV) infections and lines distinguish those with dengue fever (DF) (*n* = 5, black line) *versus* dengue haemorrhagic fever (DHF) (*n* = 5, dashed line).

In a study of CD8^+^ T cell responses to the HLA‐B57‐restricted epitope NS1_26–34_ (HTWTEQYKF) 29, we observed binding of the corresponding tetrameric antigen complex (NS1 TET) to CD8^–^ cells. As monocytes and B cells were eliminated by our gating strategy, we speculated that the NS1 TET bound a subset of NK cells. Furthermore, we hypothesized that the NS1 TET bound KIR3DL1 on NK cells, given the extensive literature describing HLA‐B57‐restricted HIV‐derived peptide ligands for this inhibitory receptor 5, 6, 7, 31, 32. Initially, we used the NS1 TET to stain PBMC obtained at a convalescent time‐point from two HLA‐B57^+^ donors in our clinical cohort. The flow cytometric gating strategy is shown in Supporting information, Fig. S1a. In parallel, we used a variant B57‐Gag_240–249_ tetramer (TW10n TET) based on a CD8^+^ T cell escape sequence (TSNLQEQIGW) of the wild‐type HIV‐derived epitope that abrogates HLA‐B57 binding to KIR3DL1*001 6. We observed substantial binding of CD8^−^ cells to the NS1 TET with minimal binding to the TW10n TET (Fig. 1a,b).

Next, we tested PBMC obtained at multiple time‐points during and after acute DENV infection from 11 HLA‐B57^+^ children, two with primary and nine with secondary DENV infection (Table 1). As our staining panel for clinical samples was developed to phenotype CD8^+^ T cells and did not include NK cell‐specific markers, we first confirmed that live lymphocytes excluding monocytes, T and B cells were predominantly NK cells. We used convalescent samples for this purpose and found that >70% of the CD3^–^CD8^–^CD14^–^CD19^–^ population comprised CD56^+^ NK cells in the majority of donors (Supporting information, Fig. S1b); these cells are referred to hereafter as the ‘NK‐enriched’ population. Although a significant proportion of NK cells can express CD8, these were excluded from our study to ensure the elimination of all T cells. This was considered important because CD3 down‐regulation during acute illness complicated the identification of T cells based solely on this marker. Evaluating the frequency of NS1 TET^+^ CD8^–^ cells in PBMC from the HLA‐B57^+^ Thai cohort, we were able to detect NS1 TET^+^ NK‐enriched cells at all time‐points tested in all donors (*n* = 10; *n* = 5 DF, *n =* 5 DHF) (Fig. 1c). The frequencies of these NS1 TET^+^ NK‐enriched cells varied over time (Fig. 1c).

**Table 1:** Clinical, virological and immunogenetic profiles of human leucocyte antigen (HLA)‐B57^+^ Thai study subjects.

Donor	Serology*	Serotype^†^	Diagnosis^‡^	KIR3DL1^§^	KIR3DS1
CHD95‐039	P	DENV‐1	DF	01502	+
CHD06‐029	P	DENV‐3	DF	01502, 01502	−
CHD05‐023	S	DENV‐1	DF	01502	+
CHD01‐018	S	DENV‐2	DF	020	+
KPP94‐037	S	DENV‐2	DF	01502,01502	−
KPP94‐041	S	DENV‐1	DHF‐3	00501	−
CHD02‐073	S	DENV‐1	DHF	00501	−
CHD01‐058	S	DENV‐2	DHF‐1	01502	+
CHD01‐050	S	DENV‐2	DHF‐3	01502	−
CHD00‐054	S	Unknown	DHF‐2	00701	+
CHD06‐092	S	DENV‐4	DHF‐2	00701,01502	+

*Primary (P) *versus* secondary (S) infection as determined by immunoglobulin (Ig)M/IgG ratios 11.

^†^Of current infection. Unknown = could not be determined.

^‡^According to WHO guidelines 1997; DF = dengue fever; DHF = dengue haemorrhagic fever (grades 1–3).

^§^KIR3DL1 subtyping.

**Figure 2:**
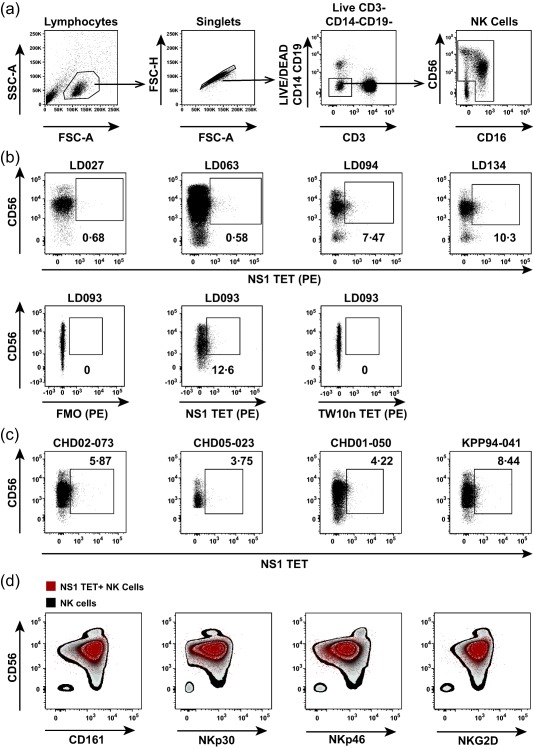
Frequencies and phenotype of NS1 tetramer (TET)^+^ natural killer (NK) cells. (a) Gating strategy to identify CD56^+^ and/or CD16^+^ NK cells. (b) Frequencies of NS1 TET^+^ NK cells in peripheral blood mononuclear cells (PBMC) from healthy KIR3DL1^+^ donors. Representative flow cytometry plots from four of 13 donors are shown on the top row. Fluorescence minus one (FMO), NS1 TET^+^ and TW10n TET^+^ NK cell frequencies in PBMC from healthy donor LD093 are shown on the bottom row. (c) Frequencies of NS1 TET^+^ NK cells in PBMC obtained from Thai study subjects 2–3 years after dengue virus (DENV) infection. (d) Overlay of NS1 TET^+^ NK cells (red dots) on the total NK cell population (zebra plot) in PBMC from a healthy KIR3DL1^+^ donor. The expression pattern of CD161, NKp30, NKp46 and NKG2D was compared between NS1 TET^+^ NK cells and the total NK cell population.

To confirm binding of the NS1 TET to NK cells, we used a staining panel with NK lineage‐specific markers (Fig. 2a,d) to analyse KIR3DL1^+^ PBMC from healthy donors and convalescent PBMC from Thai cohort subjects (Fig. 2b,c). A fluorescence minus one control excluding the NS1 TET, parallel staining with the TW10n TET and KIR3DL1 antibody labelling were used to aid gate placement for the accurate identification of NS1 TET^+^ NK cells. We observed NS1 TET^+^ NK cell populations in all donors at variable frequencies and degrees of separation. Moreover, the NS1 TET bound mainly to CD56^dim^ NK cells, which are known to express KIRs 30. Given that NK cells are highly heterogeneous, we next determined whether NS1 TET^+^ NK cells differed phenotypically from the total NK cell population. We found that NS1 TET^+^ NK cells resembled typical NK cells, in that they expressed CD161, NKp30, NKp46 and NKG2D (Fig. 2d). Thus, the NS1 TET bound archetypal CD56^dim^ NK cells.

### Binding of the NS1 TET to KIR3DL1

**Figure 3:**
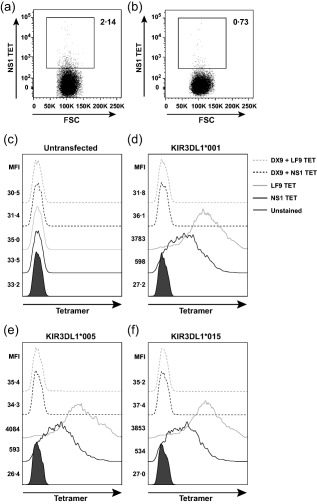
Binding of the NS1 tetramer (TET) to KIR3DL1. Using flow cytometry, (a,b) frequency of NS1 TET^+^ natural killer (NK) cells in peripheral blood mononuclear cells (PBMC) from a KIR3DL1^+^ donor before (a) and after (b) magnetic depletion of KIR3DL1^+^ cells. Data represent one of three independent experiments. (c–f) Human embryonic kidney (HEK) 293 cells were transfected with KIR3DL1 and stained with the NS1 TET (black) or the LF9 TET (grey). Histograms show NS1 TET and LF9 TET binding (solid lines) to untransfected cells (c) or cells stably transfected with KIR3DL1*001 (d), KIR3DL1*005 (e) or KIR3DL1*015 (f). Binding of the NS1 TET and the LF9 TET in the presence of a monoclonal KIR3DL1‐specific blocking antibody (DX9) is shown (dashed lines).

We speculated that binding of the NS1 TET to NK cells was mediated via the inhibitory receptor KIR3DL1. To test this possibility, we used a magnetic separation protocol to deplete PBMC of KIR3DL1^+^ cells and compared NS1 TET binding in parallel experiments with non‐depleted PBMC (Fig. 3a,b). We found that depletion of KIR3DL1^+^ cells reduced NS1 TET binding by 66%, suggesting a specific interaction between these proteins on the NK cell surface. To confirm binding of the NS1 TET to KIR3DL1 directly, we used distinct KIR3DL1‐transfected cell lines individually expressing the allotypes *001, *005 and *015, which represent the three major lineages of this inhibitory receptor 2. We observed significant binding of the NS1 TET to all three KIR3DL1 allotypes in these experiments. As expected, HLA‐B57 tetramers folded with the self‐peptide LF9 (LSSPVTKSF) also bound all three allotypes of KIR3DL1 (Fig. 3c–f) 33. Moreover, pretreatment with a KIR3DL1‐specific monoclonal antibody (DX9) blocked the binding of both tetramers to KIR3DL1 (Fig. 3c–f). Collectively, these data indicate that the NS1 TET binds KIR3DL1 on the surface of NK cells.

### Peak expression of CD38 on NS1 TET^+^ NK‐enriched cells occurs around fever day 0 and correlates with disease severity

**Figure 4:**
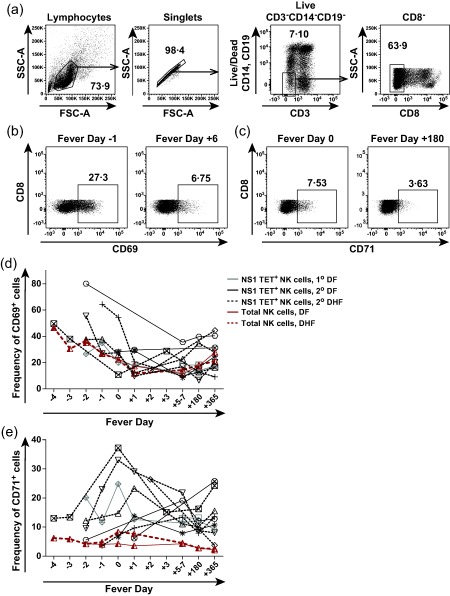
Activation of NS1 tetramer (TET)^+^ and total natural killer (NK) cells during the course of acute dengue illness. (a) Gating strategy to identify NK‐enriched cells in peripheral blood mononuclear cells (PBMC) from Thai subjects. (b) Representative flow cytometry plot depicting CD69 expression on NK‐enriched cells at fever day −1 and fever day +6 from a subject with dengue haemorrhagic fever (DHF). (c) Representative flow cytometry plot depicting CD71 expression on NK‐enriched cells at fever day 0 and fever day +180 from a subject with dengue fever (DF). (d,e) Kinetics of CD69 (d) and CD71 (e) expression on NS1 TET^+^ and total NK cells during acute dengue illness and convalescence. The average frequencies of CD69^+^ and CD71^+^ total NK‐enriched cells are shown using a solid red line for subjects with DF and a dashed red line for subjects with DHF. Symbols distinguish subjects with primary (*n* = 2, grey symbols) *versus* secondary (*n* = 8, black symbols) dengue virus (DENV) infections and lines distinguish those with DF (*n* = 5, black line) *versus* DHF (*n* = 5, dashed line).

To determine whether NS1 TET^+^ and total NK cells were activated during acute infection in HLA‐B57^+^ subjects (*n* = 2 DF 1°, *n* = 3 DF 2°, *n* = 5 DHF 2°), we assessed the expression of CD38, CD69 and CD71 on NK‐enriched populations in PBMC samples collected prior to, during and after the critical phase of DENV illness. The flow cytometric gating strategy used to identify NK‐enriched populations in these experiments is shown in Fig. 4a. Representative stainings for CD69 and CD71 expression on PBMC obtained at an acute and convalescent time‐point from a subject with DHF are shown in Fig. 4b,c. We found that CD69 expression was mildly elevated early in disease, but remained relatively high at convalescent time‐points in patients with DF and DHF (Fig. 4d). In addition, CD69 expression on NS1 TET^+^ NK cells in individual donors was similar to the expression of CD69 on total NK‐enriched cells. Peak CD71 expression occurred at fever day 0 on NS1 TET^+^ and total NK cells in many donors, but the differences were not statistically significant between patients with DF and DHF. Mean CD71 expression at acute time‐points was significantly higher in the NS1 TET^+^ NK cell population compared to total NK cells (*P* < 0·01; Fig. 4e).

**Figure 5:**
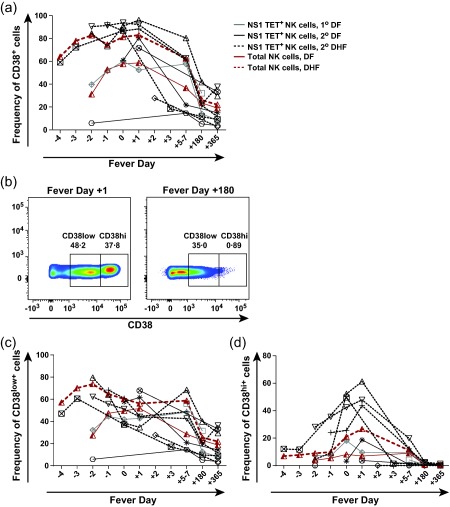
CD38 expression on NS1 tetramer (TET)^+^ and total natural killer (NK) cells during the course of acute dengue illness. (a) Kinetics of CD38 expression on NS1 TET^+^ and total NK cells during acute dengue illness and convalescence. (b) Representative flow cytometry plots depicting CD38^hi^ *versus* CD38^low^ NK cell populations at fever day +1 and fever day +180 from a subject with dengue fever (DF). (c,d) Frequencies of CD38^low^ (c) and CD38^hi^ (d) NK cell populations during acute dengue illness and convalescence. The average frequencies of CD38^hi^ and CD38^low^ total NK‐enriched cells are shown using a solid red line for subjects with DF and a dashed red line for subjects with dengue haemorrhagic fever (DHF). Symbols distinguish subjects with primary (*n* = 2, grey symbols) *versus* secondary (*n* = 8, black symbols) dengue virus (DENV) infections and lines distinguish those with DF (*n* = 5, black line) *versus* DHF (*n* = 5, dashed line).

Next, we examined CD38 expression on NK‐enriched cell populations in this HLA‐B57^+^ cohort. We found that CD38 expression was highly elevated on NK cells in PBMC during acute illness, but decreased during early convalescence and remained present on up to 40% of NK‐enriched cells 1 year after infection (Fig. 5a). More careful examination revealed that CD38 expression segregated clearly into CD38^hi^ and CD38^low^ populations on NK‐enriched cells at acute time‐points. Figure 5b shows CD38 expression on NK‐enriched cells at fever day +1 and fever day +180 in a representative donor. Frequencies of CD38^low^ cells followed the same pattern as CD69 expression on NK cells, with elevations early during infection that remained high even during convalescence (Fig. 5c). However, a different pattern was observed for CD38^hi^ cells in both the NS1 TET^+^ and total NK cell populations, with low frequencies early during acute infection becoming elevated between fever day 0 and fever day +1, then returning to baseline at 1 year post‐infection (Fig. 5d). The peak frequency of CD38^hi^ cells was observed on fever days 0 and +1 for both the total NK‐enriched and NS1 TET^+^ NK cell populations. Strikingly, very high frequencies of CD38^hi^ NS1 TET^+^ and total NK cells were observed uniquely in patients with DHF (*P* = 0·0571 compared to patients with DF).

As our original gating strategy excluded CD3^–^CD8^+^ cells in the NK‐enriched population, we further evaluated the expression of CD38, CD69 and CD71 using an inclusive approach (Supporting information, Fig. S2). Activation levels of NK‐enriched populations assessed using these markers were similar in the presence or absence of CD3^–^CD8^+^ cells. In addition, we used a quantitative polymerase chain reaction (PCR) to measure viraemia levels during early clinical illness in nine of the 11 HLA‐B57^+^ subjects. As expected, plasma virus loads were high in all donors prior to defervesence and dropped significantly as the fever dissipated (Supporting information, Fig. S3). However, no statistically significant correlations were detected between viraemia levels and CD38^hi^ NK cell frequencies (data not shown).

### Expression of KIR3DL1 on NK cells in PBMC from the HLA‐B57^+^ Thai cohort

**Figure 6:**
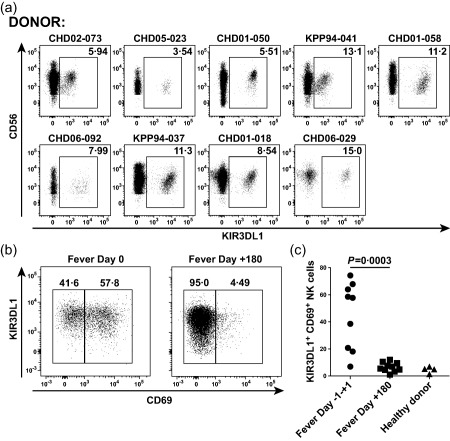
KIR3DL1 staining of natural killer (NK) cells in peripheral blood mononuclear cells (PBMC) from Thai study cohort subjects. (a) Frequencies of KIR3DL1^+^ NK cells in PBMC obtained from Thai study subjects 2–3 years after dengue virus (DENV) infection. PBMC were gated on CD56^+^ and/or CD16^+^ NK cells. Dot‐plots show CD56 versus KIR3DL1 staining. (b) Representative flow cytometry plots depicting CD69 versus KIR3DL1 expression on NK cell populations at fever day 0 and fever day +180 from a subject with dengue haemorrhagic fever (DHF). (c) Frequencies of KIR3DL1^+^CD69^+^ NK cell populations (*n* = 9) during acute dengue illness and convalescence.

To extend these findings, we examined KIR3DL1 expression on NK cells in PBMC from our Thai cohort using the KIR3DL1‐specific antibody DX9. Expression levels of KIR3DL1 are known to vary between donors 4, 30, 34, and differential expression of inhibitory KIRs can impact NK cell function significantly 35. We found substantial frequencies of KIR3DL1^+^CD56^+^ NK cells in nine of nine donors tested (Fig. 6a). The frequency of KIR3DL1 on NK cells varied from 3·5 to 15%, which is consistent with frequencies reported elsewhere 34. PBMC were not available from two subjects, but genotypical studies indicated that both were KIR3DL1^+^. The intensity of KIR3DL1 expression varied among donors, with mean fluorescence intensity (MFI) values ranging across an order of magnitude (881–7094). However, the sample size was too small to draw any conclusions regarding associations between KIR3DL1 expression, KIR3DL1 subtyping and dengue disease severity (Fig. 6a and Table 1).

Finally, we measured CD69 expression to assess NK cell activation in a limited number of PBMC samples obtained at fever day 0 (±1 day) and fever day +180. Consistent with the results presented above, we found high frequencies of KIR3DL1^+^CD69^+^ NK cells during acute infection (Fig. 6b,c). At the same time, overall KIR3DL1^+^CD56^+^ NK cell frequencies remained stable (data not shown). Collectively, these data indicate that NK cells are activated in HLA‐B57^+^ individuals during the critical phase of illness.

## Discussion

In this study, we demonstrate binding of the NK cell‐expressed inhibitory receptor KIR3DL1 to an HLA‐B57‐restricted DENV NS1‐derived peptide that also serves as a CD8^+^ T cell epitope. Direct *ex‐vivo* staining of primary human NK cells was observed with the corresponding pMHC tetramer in peripheral blood samples isolated from Thai children during and after acute DENV infection. Moreover, NS1 TET^+^ and total NK cells were activated to express CD38 during the critical phase of DENV illness only in HLA‐B57^+^ patients with DHF, suggesting that NK cell subsets may contribute to the immunopathogenesis of dengue disease. This phenotypical analysis provides the first indication of a role for KIR–HLA interactions in an acute self‐limited viral infection and suggests that innate immune receptors may determine the outcome of DENV infection alongside traditional adaptive responses 12, 14.

Interactions between MHC class I molecules and NK cell‐expressed KIRs have been associated with both beneficial and detrimental outcomes in various chronic viral infections 9 and with the development of autoimmune diseases 36. Several studies have shown that certain KIR alleles and HLA‐B loci strongly influence the rate of progression to AIDS in HIV‐infected individuals and implicate NK cells mechanistically as key determinants of viraemic control 3. The interaction between HLA‐B57 and KIR3DL1 has been studied extensively in this context. For example, Fadda *et al*. showed that naturally occurring single amino acid escape mutations in HLA‐B57‐restricted HIV‐derived CD8^+^ T cell epitopes could abolish KIR3DL1 binding completely 6, 33. Similarly, the interaction between B57‐NS1_26–34_ and KIR3DL1 may represent a novel strategy by which DENV evades NK cell‐mediated immunity. Functional studies are in progress to address this possibility. Polyfunctional assays with HLA‐B57^+^ NK sensitive targets are critical to determine whether the DENV NS1 peptide can modulate NK cell function and are an active area of research in the laboratory.

In longitudinal phenotypical analyses, we found that CD69 expression on NK‐enriched cells was elevated early during acute infection. In contrast, CD71^+^ and CD38^hi^ NK cells were rare at this time‐point and became more prevalent later, with peak frequencies around fever day 0 in several donors. The emergence of abundant CD38^hi^ NK cells coincided with peak CD8^+^ T cell activation in this cohort and the critical period for plasma leakage and thrombocytopenia in patients with DHF 29. Moreover, CD38^hi^ expression on NK‐enriched cells differed substantially between patients with mild (DF) and severe (DHF) dengue disease. These distinct activation patterns may preclude the identification of clinically relevant biomarkers in acute DENV infection.

The late activation of NK cells could be a consequence of the cytokine storm associated with DHF. In this scenario, NS1 TET^+^ (and therefore KIR3DL1^+^) NK cells might be driven to expand preferentially in HLA‐B57^+^ hosts due to more efficient licensing. Alternatively, NS1 TET^+^ cells may represent a subset of NK cells that are restrained early in infection due to interactions between B57‐NS1_26–34_ and KIR3DL1. As flaviviruses are known to up‐regulate MHC class I 37, we propose that the increased expression of HLA‐B57 on target cells early in infection augments NS1 peptide presentation during the acute viraemic phase, thus enhancing KIR3DL1 interactions and maintaining NK cell inhibition. As viral titres fall and MHC class I expression returns to normal during defervescence, B57‐NS1_26–34_ levels will also wane and allow ‘retuned’ NK cells to respond vigorously.

Despite collection over a 15‐year time‐period, we were only able to enrol a total of 15 HLA‐B57^+^ donors due to the low frequency of this allele in Thailand. This limitation impacted the power of our study and the differences in CD38^hi^ expression did not quite achieve statistical significance (*P =* 0·0571). In addition, the relative rarity of HLA‐B*57 may confine the clinical relevance of DENV NS1_26–34_ in the Thai population. The fact that not all HLA‐B57^+^ KIR3DL1^+^ individuals develop DHF suggests the involvement of additional regulatory loops 38. Given the stochastic expression of KIRs, different individuals will co‐express different combinations of inhibitory and activating receptors within the KIR3DL1^+^ NK cell subset. This constellation of receptor/ligand interactions will probably contribute to differential effects on NK cell function. In addition, elevated levels of cytokines known to be up‐regulated in patients with dengue will almost certainly influence the quality of NK cell and T cell responses. It is notable in this respect that the DENV envelope (E) protein interacts directly with the NK cell activating receptor NKp44 39.

As with most clinical studies of dengue, the delay between initial viral infection and presentation to the clinic or hospital prevented a very early assessment of NK cell activation in this cohort. A rapid NK cell response that leads to pathogen elimination may reduce the levels of antigen available for presentation, thereby potentially impairing the development of memory T cell populations. Indeed, NK cells have been implicated in the regulation of T cell immunity during viral infections, purportedly acting to prevent pathological responses by attenuating T cell activation in the presence of high viral loads 40, 41, 42. In this study, we found delayed activation of NK cells in HLA‐B57^+^ KIR3DL1^+^ donors, which could hamper the development of protective memory T cell responses to DENV. This regulatory activity of NK cells could explain the modest CD8^+^ T cell responses directed against this highly conserved NS1 epitope in secondary DENV infections 29.

In conclusion, our findings suggest that NK cell subsets play a role in the development of adverse immune responses associated with DHF in the context of HLA‐B57. Further studies are warranted to identify determinative KIR–HLA interactions in other acute self‐limited viral infections.

## Author contributions

E. T., A. M. and A. L. R. conceived and designed the experiments and wrote the manuscript text. E. T., G. O. C. and M. W. performed the experiments. E. T. and A. M. analysed the data. E. T., A. M. and D. A. P. prepared the figures. M. C., S. J. T., S. K., I. K. Y., A. N., A. S. and S. G. enrolled patients and collected samples. C. C., E. G., D. A. P., M C., G. A. and D. W. M. contributed reagents, protocols. H. A. F. S. provided HLA typing data. All authors reviewed the manuscript and agree with the results and conclusions.

## Disclosure

The authors declare no commercial or financial disclosures.

## Supporting information

Additional Supporting information may be found in the online version of this article at the publisher's web‐site:

**Fig. S1**. Frequencies of natural killer (NK) cells in the CD3^–^CD8^–^CD14^–^CD19^–^ gate. (a) Gating strategy to identify CD3^–^CD8^–^CD14^–^CD19^–^ cells. Cells were first selected within the lymphocyte gate as defined by forward‐ and side‐scatter profiles. Singlets were then identified and live CD3^–^CD14^–^CD19^–^ cells were selected in a dump (LIVE/DEAD^®^ Green with αCD14 and αCD19) *versus* CD3 bivariate plot. CD8^–^ cells were gated within this population. (b) Frequencies of CD56^+^ and/or CD16^+^ NK cells in peripheral blood mononuclear cells (PBMCs) collected from Thai cohort subjects 2 years after acute dengue virus (DENV) infection. Plots are gated on live CD3^–^CD8^–^CD14^–^CD19^–^ cells.
**Fig. S2**. Activation of NS1 TET^+^ and total natural killer (NK) cells over the course of acute dengue illness. Kinetics of CD69 (a), CD71 (b), total CD38 (c), CD38low (d) and CD38^hi^ (e) expression on NS1 TET^+^ and total NK cells during acute dengue illness and convalescence. The average frequencies of CD69^+^, CD71^+^, total CD38^+^, CD38^low^, and CD38^hi^ total NK‐enriched cells are shown using a solid red line for subjects with dengue fever (DF) and a dashed red line for subjects with dengue haemorrhagic fever (DHF). Symbols distinguish subjects with primary (*n* = 2, grey symbols) versus secondary (*n* = 8, black symbols) dengue virus (DENV) infections and lines distinguish those with DF (*n* = 5, black line) *versus* DHF (*n* = 5, dashed line).
**Fig. S3**. Magnitude of dengue virus (DENV) viraemia by day of illness. Levels of DENV genome equivalent (GE) cDNA (copies/ml) were determined in serial plasma samples from human leucocyte antigen (HLA)‐B57^+^ patients. Symbols denote individual subjects and lines distinguish those with dengue fever (DF) (*n* = 4, black line) *versus* dengue haemorrhagic fever (DHF) (*n* = 5, dashed line).
**Table S1**. Antibodies used for flow cytometry studies.
