# Supplementary material for: Interaction of a dengue virus NS1‐derived peptide with the inhibitory receptor KIR3DL1 on natural killer cells
Source: Clin Exp Immunol. 2015 Nov 24;183(3):419–30. doi: 10.1111/cei.12722 (PMC4750593; doi:10.1111/cei.12722)
Supplement: Supplementary file 1 — Fig. S1. Frequencies of natural killer (NK) cells in the CD3–CD8–CD14–CD19– gate. (a) Gating strategy to identify CD3–CD8–CD14–CD19– cells. Cells were first selected within the lymphocyte gate as defined by forward‐ and side‐scatter profiles. Singlets were then identified and live CD3–CD14–CD19– cells were selected in a dump (LIVE/DEAD® Green with αCD14 and αCD19) versus CD3 bivariate plot. CD8– cells were gated within this population. (b) Frequencies of CD56+ and/or CD16+ NK cells in peripheral blood mononuclear cells (PBMCs) collected from Thai cohort subjects 2 years after acute dengue virus (DENV) infection. Plots are gated on live CD3–CD8–CD14–CD19– cells. Fig. S2. Activation of NS1 TET+ and total natural killer (NK) cells over the course of acute dengue illness. Kinetics of CD69 (a), CD71 (b), total CD38 (c), CD38low (d) and CD38hi (e) expression on NS1 TET+ and total NK cells during acute dengue illness and convalescence. The average frequencies of CD69+, CD71+, total CD38+, CD38low, and CD38hi total NK‐enriched cells are shown using a solid red line for subjects with dengue fever (DF) and a dashed red line for subjects with dengue haemorrhagic fever (DHF). Symbols distinguish subjects with primary (n = 2, grey symbols) versus secondary (n = 8, black symbols) dengue virus (DENV) infections and lines distinguish those with DF (n = 5, black line) versus DHF (n = 5, dashed line). Fig. S3. Magnitude of dengue virus (DENV) viraemia by day of illness. Levels of DENV genome equivalent (GE) cDNA (copies/ml) were determined in serial plasma samples from human leucocyte antigen (HLA)‐B57+ patients. Symbols denote individual subjects and lines distinguish those with dengue fever (DF) (n = 4, black line) versus dengue haemorrhagic fever (DHF) (n = 5, dashed line). Table S1. Antibodies used for flow cytometry studies. [file CEI-183-419-s001.pdf]

Supplemental Figure 1

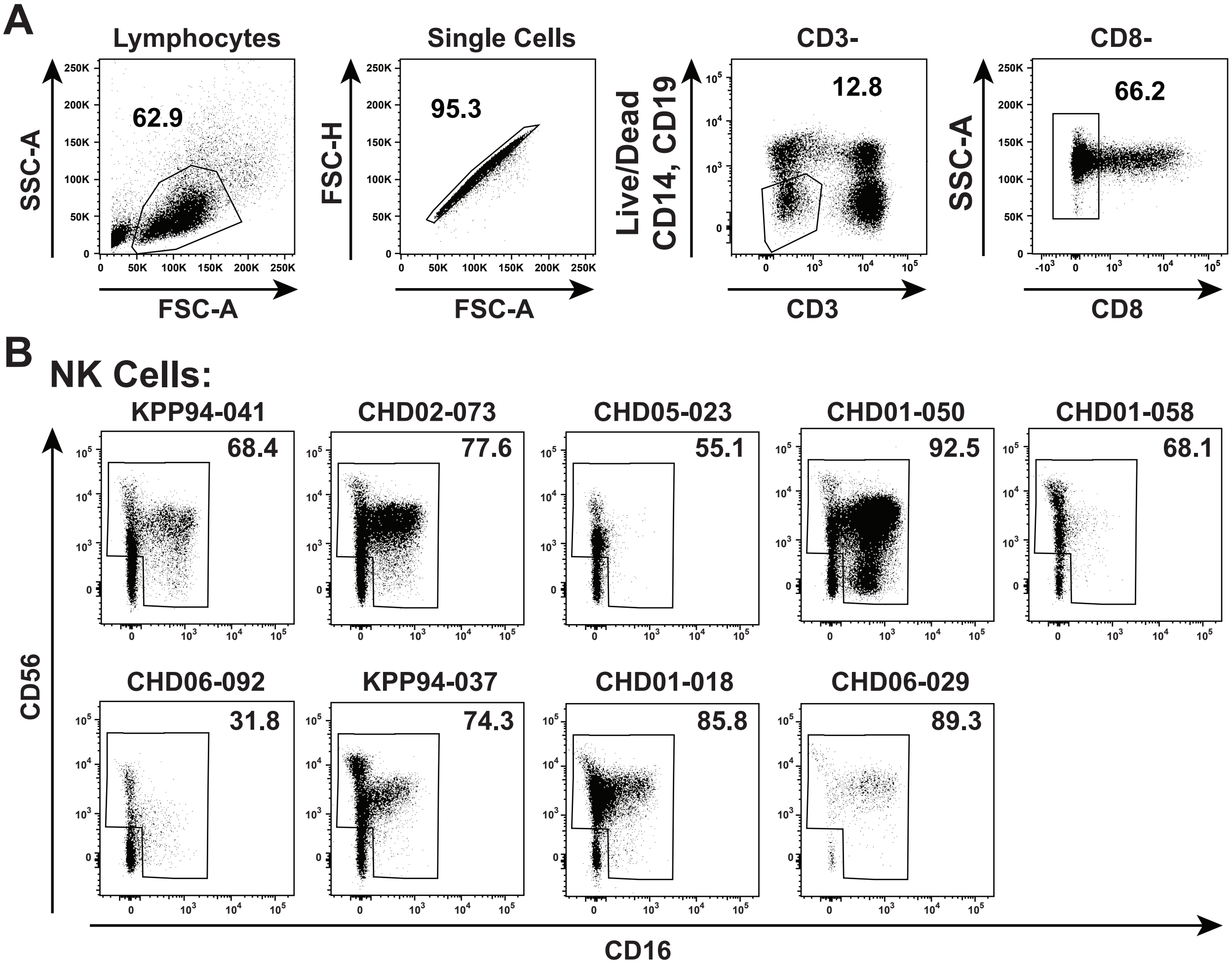

Supplemental Figure S1. Frequencies of NK cells in the CD3$^-$CD8$^-$CD14$^-$CD19$^-$ gate. (A) Gating strategy to identify CD3$^-$CD8$^-$CD14$^-$CD19$^-$ cells. Cells were first selected within the lymphocyte gate as defined by forward and side scatter profiles. Singlets were then identified and live CD3$^-$CD14$^-$CD19$^-$ cells were selected in a dump (LIVE/DEAD® Green with αCD14 and αCD19) versus CD3 bivariate plot. CD8$^-$ cells were gated within this population. (B) Frequencies of CD56$^+$ and/or CD16$^+$ NK cells in PBMCs collected from Thai cohort subjects 2 years after acute DENV infection. Plots are gated on live CD3$^-$CD8$^-$CD14$^-$CD19$^-$ cells.

Supplemental Figure 2

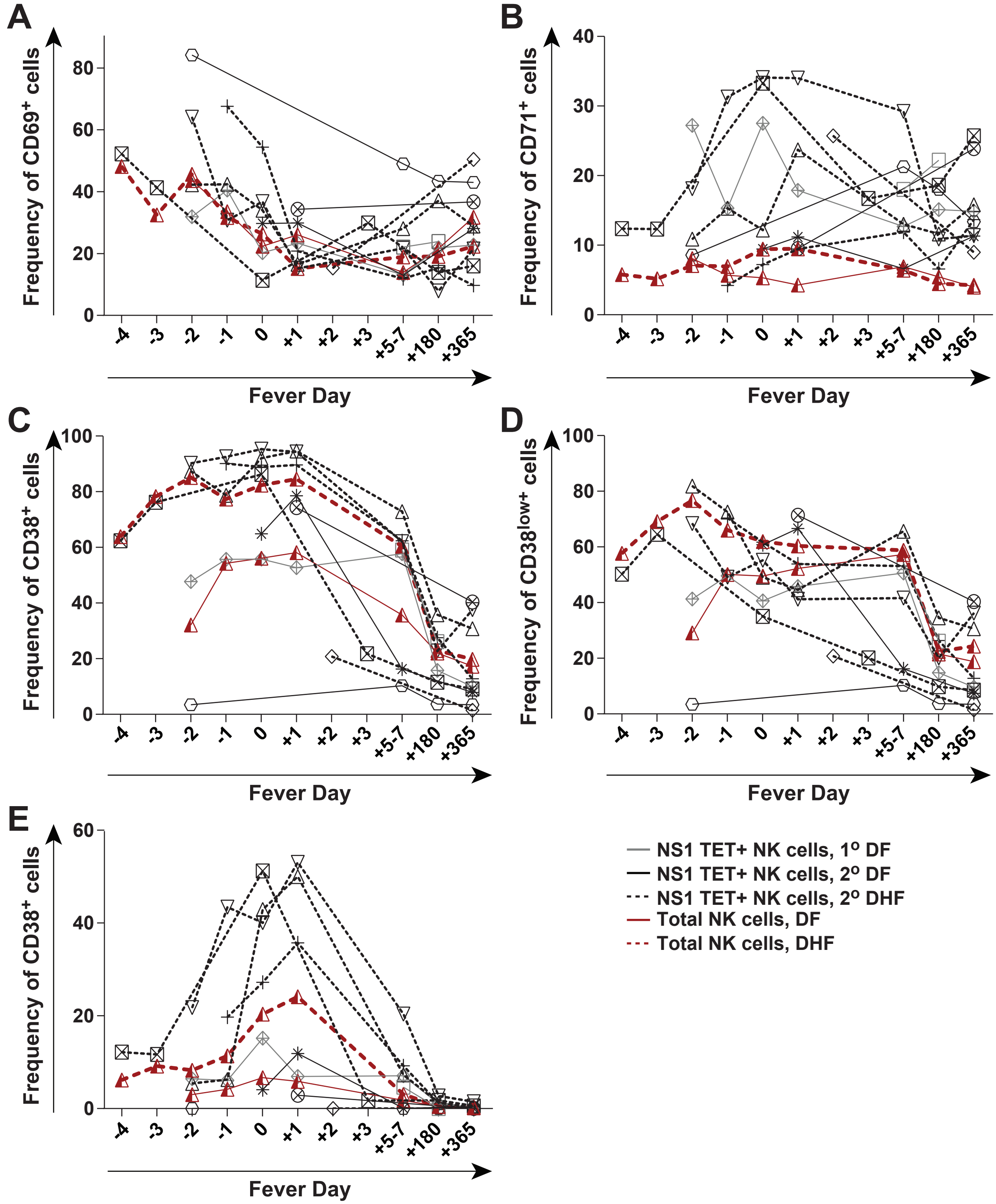

Supplemental Figure S2. Activation of NS1 TET+ and total NK cells over the course of acute dengue illness. Kinetics of CD69 (A), CD71 (B), total CD38 (C), $CD38^{low}$ (D), and $CD38^{hi}$ (E) expression on NS1 TET+ and total NK cells during acute dengue illness and convalescence. The average frequencies of CD69+, CD71+, total CD38+, $CD38^{low}$, and $CD38^{hi}$ total NK-enriched cells are shown using a solid red line for subjects with DF and a dashed red line for subjects with DHF. Symbols distinguish subjects with primary (*n=2*, grey symbols) versus secondary (*n=8*, black symbols) DENV infections and lines distinguish those with DF (*n=5*, black line) versus DHF (*n=5*, dashed line).

Supplemental Figure 3

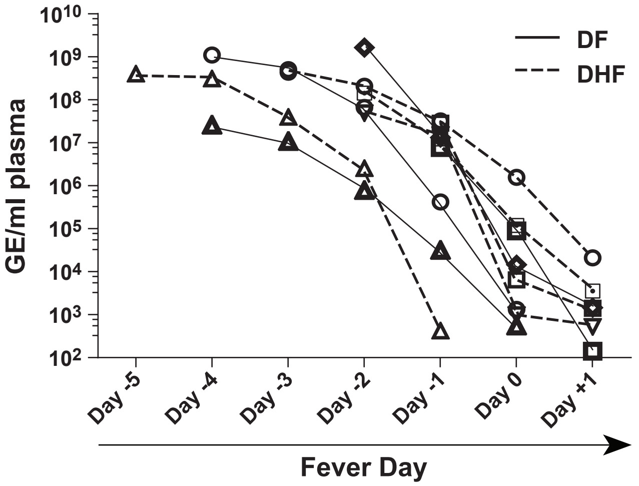

Supplemental Figure S3. Magnitude of DENV viremia by day of illness. Levels of DENV genome equivalent (GE) cDNA (copies/mL) were determined in serial plasma samples from HLA-B57$^+$ patients. Symbols denote individual subjects and lines distinguish those with DF (*n=4*, black line) versus DHF (*n=5*, dashed line).

SUPPLEMENTAL TABLE 1: Antibodies used for flow cytometry studies

Marker	Clone	Manufacturer	Fluorochrome
**CD3**	UCHT1	BD Biosciences	V500
		BioLegend	BV510
	OKT3	BioLegend	BV510
**CD8**	SK1	Invitrogen	PE-AlexaFluor610
**CD45RA**	HI100	BD Pharmingen	APC-H7
**CCR7**	150503	BD Horizon	V450
**CD69**	CH/4	Invitrogen	PE-Cy5.5
	FN50	BioLegend	BV650
**CD38**	HB7	eBioscience	eFluor®650NC
**CD57**	HCD57	BioLegend	PerCP/Cy5.5 (Lightening Link)
**CD71**	OKT9	eBioscience	PE-Cy7 (Lightening Link)
	CY1G4	BioLegend	APC
**CD28**	CD28.2	BioLegend	AlexaFluor700
**CD56**	B159	BD Biosciences	AlexaFluor700
**CD19**	HIB19	BD Biosciences	FITC
**CD14**	HCD14	BioLegend	FITC
**CD56**	HCD56	BioLegend	BV711
**CD16**	3G8	BD Horizon	APC-H7
**NKp30**	P30-15	BioLegend	APC
**NKp46**	9E2	BioLegend	BV421
**CD161**	HP-3G10	BioLegend	BV605
**NKG2D**	1D11	BD Biosciences	PE-CF594
**KIR3DL1**	DX9	Beckman Coulter	PE
		BioLegend	PE
**KIR3DL1/S1**	Z27	Beckman Coulter	APC
**HLA-A,B,C**	W6/32	BD Biosciences	APC, PE, FITC
		BioLegend	PE
**HLA-B57**	BIH0243	One Lambda	PE-NeutrAvidin
